# A Novel Member of the Trehalose Transporter Family Functions as an H^+^-Dependent Trehalose Transporter in the Reabsorption of Trehalose in Malpighian Tubules

**DOI:** 10.3389/fphys.2012.00290

**Published:** 2012-07-25

**Authors:** Shingo Kikuta, Yuka Hagiwara-Komoda, Hiroaki Noda, Takahiro Kikawada

**Affiliations:** ^1^National Institute of Agrobiological SciencesTsukuba, Ibaraki, Japan; ^2^Department of Integrated Biosciences, Graduate School of Frontier Sciences, The University of TokyoKashiwa, Chiba, Japan

**Keywords:** trehalose, sugar reabsorption, Malpighian tubules, proton-dependent transporter

## Abstract

In insects, Malpighian tubules are functionally analogous to mammalian kidneys in that they not only are essential to excrete waste molecules into the lumen but also are responsible for the reabsorption of indispensable molecules, such as sugars, from the lumen to the principal cells. Among sugars, the disaccharide trehalose is highly important to insects because it is the main hemolymph sugar to serve as a source of energy and carbon. The trehalose transporter TRET1 participates in the transfer of newly synthesized trehalose from the fat body across the cellular membrane into the hemolymph. Although transport proteins must play a pivotal role in the reabsorption of trehalose in Malpighian tubules, the molecular context underlying this process remains obscure. Previously, we identified a *Tret1* homolog (*Nlst8*) that is expressed principally in the Malpighian tubules of the brown planthopper (BPH). Here, we used the *Xenopus* oocyte expression system to show that NlST8 exerts trehalose transport activity that is elevated under low pH conditions. These functional assays indicate that *Nlst8* encodes a proton-dependent trehalose transporter (H-TRET1). To examine the involvement of *Nlst8* in trehalose reabsorption, we analyzed the sugar composition of honeydew by using BPH with RNAi gene silencing. Trehalose was detected in the honeydew as waste excreted from *Nlst8*-dsRNA-injected BPH under hyperglycemic conditions. However, trehalose was not expelled from *GFP*-dsRNA-injected BPH even under hyperglycemic conditions. We conclude that NlST8 could participate in trehalose reabsorption driven by a H^+^ gradient from the lumen to the principal cells of the Malpighian tubules.

## Introduction

Excretory organs, kidneys in vertebrates and the Malpighian tubules in invertebrates, are essential to discharge waste, such as small molecules and excess salt, into the renal lumen. For small molecules in particular, this excretion step occurs through non-selective filtration, which means that the molecules required by living organisms must be retrieved from the waste. Therefore, another function of excretory organs is to reabsorb indispensable molecules, including sugars, amino acids, and water, via dedicated transporters located in the cellular membrane. For example, in mammals, sodium-glucose co-transporter 2 (SGLT2), which is expressed in the apical membrane of kidney cells facing the lumen, has a pivotal role in glucose reabsorption driven by electrochemical membrane potentials (Wright, 2001).

In most insects, trehalose, a disaccharide composed of two glucose molecules linked by an α-1,1-bond, is the main hemolymph sugar; it acts as a nutrient source and as a protectant against harsh conditions, such as desiccation, heat, and cold (Crowe et al., 1998; Arrese and Soulages, 2010). In *Locusta migratoria* and *Schistocerca gregaria*, trehalose is degraded by trehalase to be utilized as the primary energy source for flight (Vaandrager et al., 1989; Becker et al., 1996). In the larvae of the sleeping chironomid, *Polypedilum vanderplanki*, trehalose is intensively synthesized under dehydrating conditions and eventually vitrified (Mitsumasu et al., 2010); it thus acts as an anhydroprotectant (Watanabe et al., 2002; Sakurai et al., 2008). Trehalose produced in the fat body is exported to the hemolymph (Wyatt, 1961; Mitsumasu et al., 2010), where it may passively and non-specifically leak into the tubule lumens and be excreted as waste (Knowles, 1975). Although trehalose was thought to be reabsorbed from the lumen to the principal cells of the tubules (Knowles, 1975; Jarial and Kelly-Worden, 2011), the molecular basis of this process has remained unknown.

Sugar transporters have essential roles in the appropriate distribution of carbohydrates throughout the body (Mueckler, 1994). They are typically categorized in two groups: (i) secondary active membrane transporters, which promote the uphill permeation of sugars driven by electrochemical gradients of Na^+^ or H^+^ ions across the cellular membranes, and (ii) facilitative sugar transporters, which enable sugars to flow across membranes down concentration gradients (Wood and Trayhurn, 2003). Until now, trehalose transporters have been identified from yeasts and insects (Stambuk et al., 1998; Kikawada et al., 2007). *Saccharomyces cerevisiae* possesses the α-glucoside transporter AGT1, which promotes uptake of disaccharides, including trehalose, sucrose, and maltose, via an electrochemical proton gradient, which suggests that AGT1 belongs to the group of secondary active transporters (Han et al., 1995). Thus, AGT1 acts as an H^+^-dependent trehalose transporter for the uptake of low-level trehalose as a nutrient from culture medium under low pH conditions. Insects have a facilitative trehalose transporter, TRET1, which seems to be responsible for the regulation of trehalose levels in the hemolymph (Kikawada et al., 2007; Kanamori et al., 2010). Secondary active transporters rather than facilitative transporters may mediate reabsorption of trehalose in Malpighian tubules because the passively diffused trehalose concentration in the lumen should be lower than the concentration in hemolymph. Yet, secondary active transporters for trehalose in multicellular organisms, including insects, have not been reported.

It is difficult to predict the characteristics of sugar transporters based solely on their amino acid sequences because only subtle difference exists between proton-dependent sugar transporters and facilitative sugar transporters in the major facilitator superfamily (MFS; Pao et al., 1998). In plants, for instance, the sucrose transporter LjSUT4 from *Lotus japonicus* shares 73% identity at the amino acid level with PsSUF4 from *Pisum sativum* (Zhou et al., 2007), although LjSUT4 is an H^+^ dependent transporter and PsSUF4 is a facilitative transporter for sucrose (Zhou et al., 2007; Reinders et al., 2008). These results suggest that proton-dependent trehalose transporters may also reside in the TRET1 family in insects. Hence, the amino acid sequence of the H^+^-dependent trehalose transporter should be similar to that of the facilitative trehalose transporter, relative to other sugar transporters.

**Figure A1:**
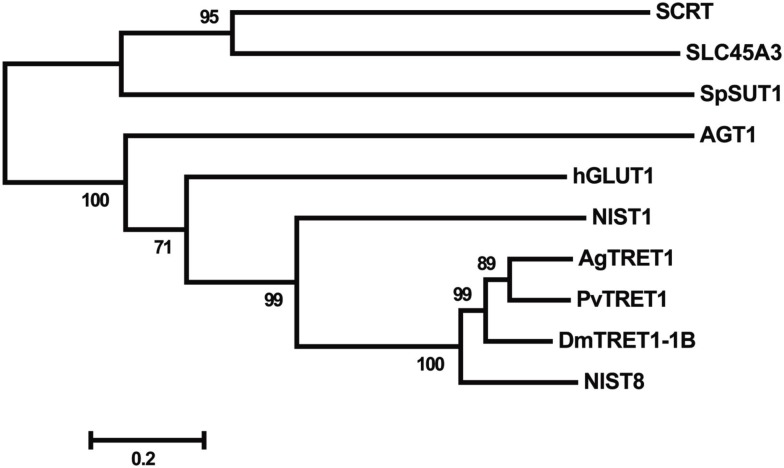
**Phylogenetic analysis of the deduced amino acid sequences of putative trehalose transporters**. A rooted tree was constructed with the neighbor-joining method using MEGA ver. 5 (Tamura et al., 2011), and the tree was tested with a bootstrap analysis of 1,000 replications. The insect trehalose transporters were: NlST8 from *N. lugens* (NCBI accession number: AB550001), AgTRET1 from *A. gambiae* (AB369548), PvTRET1 from *P. vanderplanki* (AB272983), and DmTRET 1-1B from *D. melanogaster* (AB369553). The glucose transporter NlST1 of *N. lugens* (AB549994) was applied as an out-group of the trehalose transporter family. SCRT (CG4484), derived from *D. melangaster*, is a highly similar protein that belongs to the mammalian solute carrier family 45 (SLC45) and is an H^+^-sucrose co-transporter. The other amino acid sequences, AGT1 of *S. cerevisiae*, SpSUT1 of *Schizosaccharomyces pombe* (NP_594387.1), human SLC45A3 (NP_149093), and human hGLUT1 (NP_006507.2) were obtained from the NCBI public database.

The TRET1 family constitutes a mono-clade among the insect sugar transporters (Figure A1 in Appendix; Kikuta et al., 2010). Among the TRET1 family, NlST8, which was isolated from the brown planthopper (BPH) *Nilaparvata lugens*, a rice plant pest (Kikuta et al., 2010), could be a transporter in trehalose reabsorption, because NlST8 is mainly expressed in Malpighian tubules (Kikuta et al., 2010). Here, we investigated the transport activity and physiological roles of NlST8 by using the *Xenopus* oocyte expression system and the technique of RNAi gene silencing, respectively. Our results indicate that NlST8 is a proton-dependent TRET1 with a role in trehalose reabsorption in Malpighian tubules.

## Results

### Subcellular localization of NLST8 in *Xenopus* oocytes

**Figure 1:**
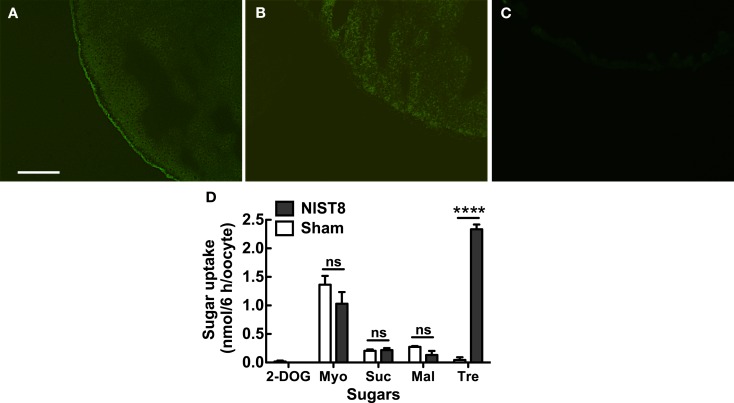
**Functional analyses of NlST8-expressing *Xenopus* oocyte**. Localization of NlST8 in the membrane of the *Xenopus* oocyte as detected by use of an AcGFP1 fusion protein. **(A)** NlST8::AcGFP1 fluorescence was observed in *Xenopus* oocytes. **(B)** *AcGFP1* cRNA injection as a control. **(C)** Sham as a negative control. Scale bar, 20 μm. **(D)** Sugar uptake analyses of NlST8 by using HPLC. Transporters were expressed in the cellular membrane of *Xenopus* oocytes by injecting the cRNA of *Nlst8*. 2-DOG, 2-deoxy-glucose; Myo, *myo*-inositol; Suc, sucrose; Mal, maltose; Tre, Trehalose. Sham is a negative control. Sugars were used at a concentration of 105 mM in MBS buffer. Ten oocytes were analyzed in each assay. Error bars represent the standard error (*n* = 3). Statistical significance was determined by using Student’s *t*-test in each assay. “ns” indicates no significant difference; asterisks indicate a significant difference (****P < 0.0001). *Myo*-inositol: *P* = 0.2626, sucrose: *P* = 0.8057, maltose: *P* = 0.1241, and trehalose: *P* < 0.0001.

To determine whether NIST8 is a membrane-bound protein, we examined its subcellular localization by using either a GFP-fusion protein, NIST8::AcGFP1, or GFP alone in *Xenopus* oocytes. Fluorescence of the fusion protein was principally detected in the cellular membrane (Figure 1A) but not in the cellular membrane of oocytes injected with *AcGFP1* capped RNA (cRNA) only (Figure 1B). Of course, no fluorescence was detected in a sham control (Figure 1C). These results indicate that *Nlst8* encodes a membrane-bound protein.

### NLST8 is a trehalose transporter

To explore substrates for NlST8, we conducted a sugar uptake assay with trehalose and other carbohydrates, including *myo*-inositol, sucrose, maltose, and 2-deoxy-d-glucose (2-DOG; Figure 1D) using the NlST8-expressing *Xenopus* oocytes. Of the carbohydrates tested, only trehalose was significantly taken up by oocytes (*P* < 0.0001) compared with a sham control, indicating that NlST8 possessed trehalose transport activity.

### Trehalose uptake via NLST8 is driven by a proton gradient

**Figure 2:**
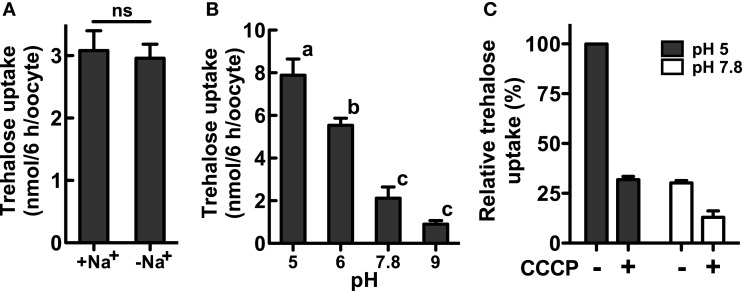
**Trehalose uptake via NlST8 is driven at low pH**. Ten oocytes were analyzed in each assay; error bars represent the standard error (*n* = 3). The trehalose concentration used was 105 mM. **(A)** Trehalose uptake was estimated under Na^+^-free conditions. Statistical significance was determined by using Student’s *t*-test in the assays; “ns” indicates no significant difference (*P* = 0.7686). **(B)** Trehalose uptake was examined under various pH conditions. Statistical analyses were performed by using one-way ANOVA before Tukey’s multiple comparison tests. Columns labeled with the same letters indicate no significant difference (*P* > 0.05). **(C)** Trehalose uptake in the presence of the protonophore CCCP (50 μM). Gray bars show trehalose uptake at pH 5 in 105 mM trehalose solution, and white bars show trehalose uptake at pH 7.8.

**Figure A2:**
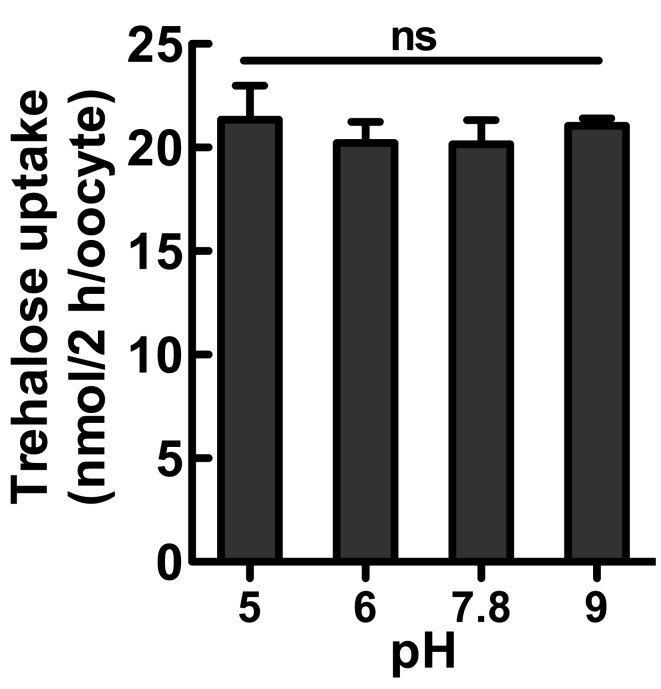
**Trehalose uptake into *Xenopus* oocytes via PvTRET1 at various pH values**. Ten oocytes were analyzed in each assay; error bars represent the standard error (*n* = 3). The PvTRET1-expressing oocytes were incubated in 105 mM trehalose solutions. Statistical significance was determined by using one-way ANOVA. “ns” indicates no significant difference (*P* = 0.845).

**Figure A3:**
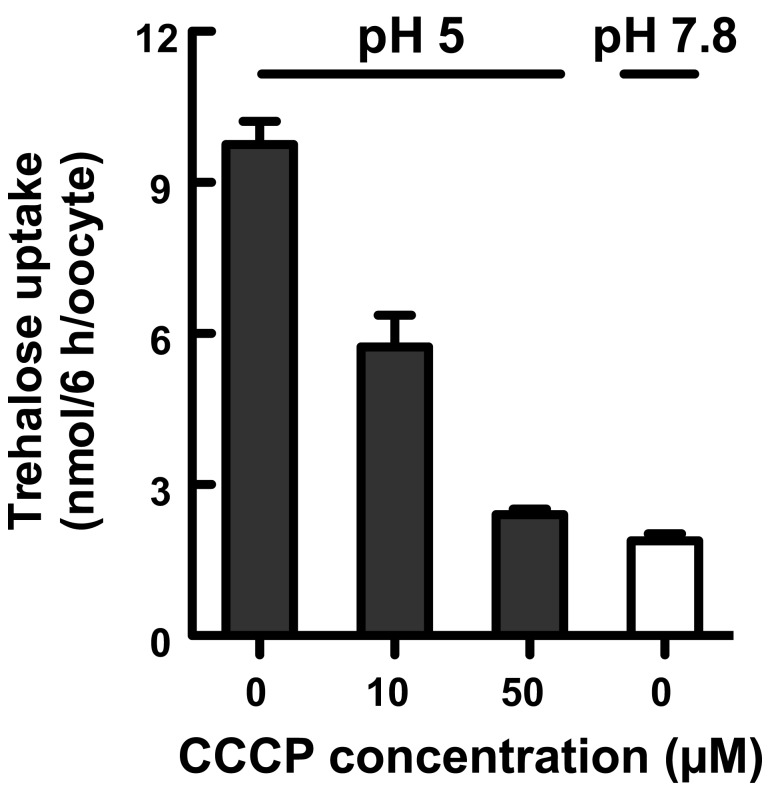
**Trehalose transport via NlST8 is proton-dependent**. Trehalose uptake into oocytes expressing NlST8 was examined at pH 5 with increasing concentrations of the protonophore CCCP (0, 10, and 50 μM). Trehalose uptake at pH 7.8 is shown as a negative control. Ten oocytes were analyzed in each assay; error bars represent the standard error (*n* = 3). The trehalose concentration in the buffer was 105 mM.

We investigated the biochemical properties of NlST8’s transport activity for trehalose. First, we analyzed trehalose uptake via NlST8 under Na^+^-free conditions. The result indicated that trehalose uptake by NlST8 was Na^+^-independent (Figure 2A). The pH dependency of NlST8 for trehalose uptake was also examined under various pH conditions, ranging from 5.0 to 9.0. Trehalose transport activity of NlST8 was clearly increased at the lower pH values (Figure 2B). At pH 5 in MES [2-(*N*-morpholino) ethanesulfonic acid] buffer, trehalose uptake was approximately four times that at pH 7.8. In contrast, the activity of the facilitated trehalose transporter PvTRET1 was pH-independent, as reported previously (Figure A2 in Appendix; Kikawada et al., 2007). A protonophore, carbonyl cyanide *m*-chlorophenyl hydrazone (CCCP), inhibited trehalose uptake via NlST8 even at pH 5 (Figure A3 in Appendix; Figure 2C). Protonophores allow protons to selectively cross the cellular membranes, resulting in disruption of proton gradient across the membranes. Thus, these results suggest that trehalose uptake by NlST8 is probably driven via a proton gradient across the membrane.

### Trehalose transport kinetics for NLST8

**Figure 3:**
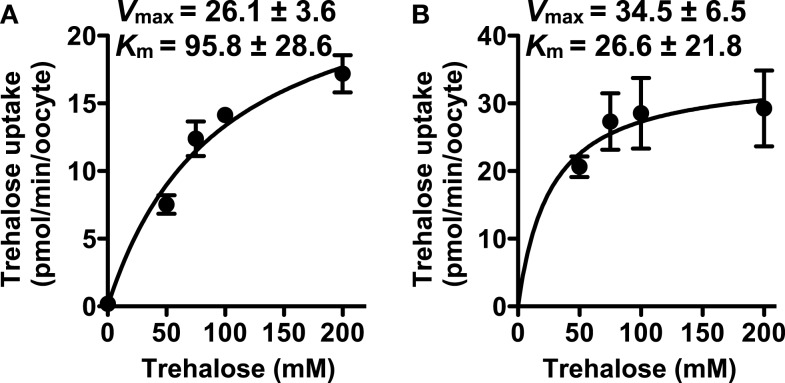
**Analyses of the kinetics of NlST8 for trehalose**. Ten oocytes were analyzed in each assay; error bars represent the standard error (*n* = 3). **(A)** Oocytes expressing NlST8 were incubated with various trehalose concentrations for 3 h at pH 7.8. **(B)** Oocytes expressing NlST8 were incubated for 1 h with various trehalose concentrations at pH 5. Data were fitted to the Michaelis–Menten equation.

The kinetics values of NlST8 for trehalose were determined at either pH 7.8 (normal physiological conditions for *Xenopus* oocytes) or pH 5 (low pH condition). The values at pH 7.8 were *K*_m_ = 95.8 ± 28.6 and *V*_max_ = 26.1 ± 3.6 (Figure 3A), whereas the values at pH 5 were *K*_m_ = 26.6 ± 21.8 and *V*_max_ = 34.5 ± 6.5 (Figure 3B). Namely, lower pH conditions conduced to higher affinity and capacity for trehalose transport activity of NlST8. This kinetic analysis supported that NlST8 was likely to act as a proton-dependent trehalose transporter, i.e., an H^+^-trehalose symporter.

### NLST8 is involved in trehalose reabsorption

**Figure 4:**
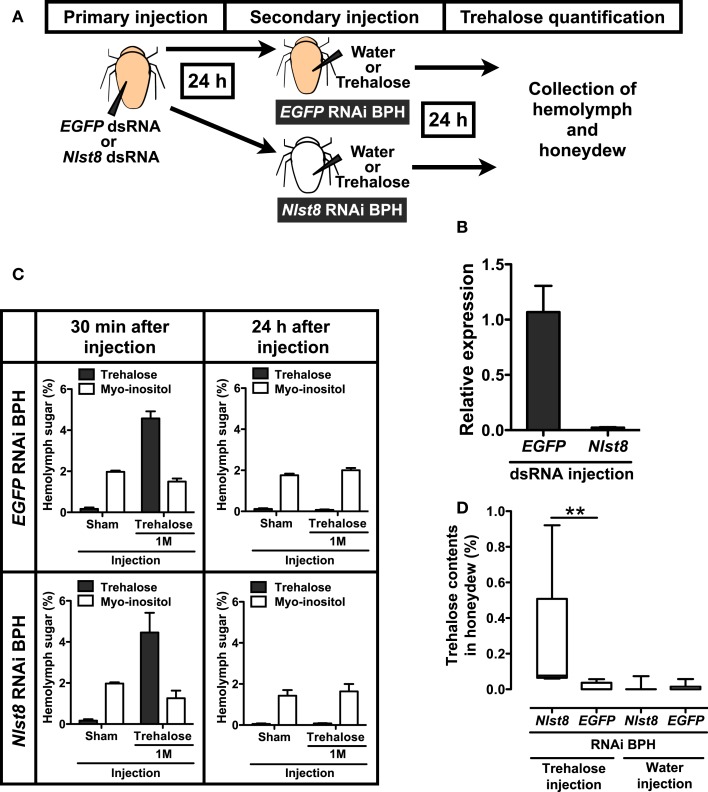
**Reabsorption of trehalose in BPH**. **(A)** Experimental scheme of hemolymph and honeydew analysis. RNAi techniques and quantification of sugars in hemolymph and honeydew are described in the Appendix. Briefly, BPH female adults were injected with dsRNA solution in the segment between the thorax and abdomen and incubated for 24 h to suppress gene expression. Each *EGFP1* or *Nlst8* RNAi BPH was then injected with 50 nl of water or trehalose in the same segment and incubated for 24 h in a Parafilm sachet. Finally, sugars in BPH hemolymph or in honeydew were analyzed by using HPLC. Sugar contents are represented as percentages (mg/100 μl). **(B)** Effect of RNAi on the gene expression levels of *Nlst8* at 48 h after injection. *EGFP1*-dsRNA served as a negative control. Error bars represent the standard error (*n* = 3). **(C)** Hemolymph sugar contents following injection of water or 1 M trehalose into *EGFP1* (upper) or *Nlst8* (lower) RNAi BPH. Sugar contents were analyzed at 30 min after injection (left), and at 24 h after injection (right). **(D)** Quantifications of trehalose in BPH honeydew. Statistical analyses were performed by using the Kruskal–Wallis test before Dunn’s multiple comparison tests. The asterisk indicates a significant difference (*P* = 0.0023).

**Figure A4:**
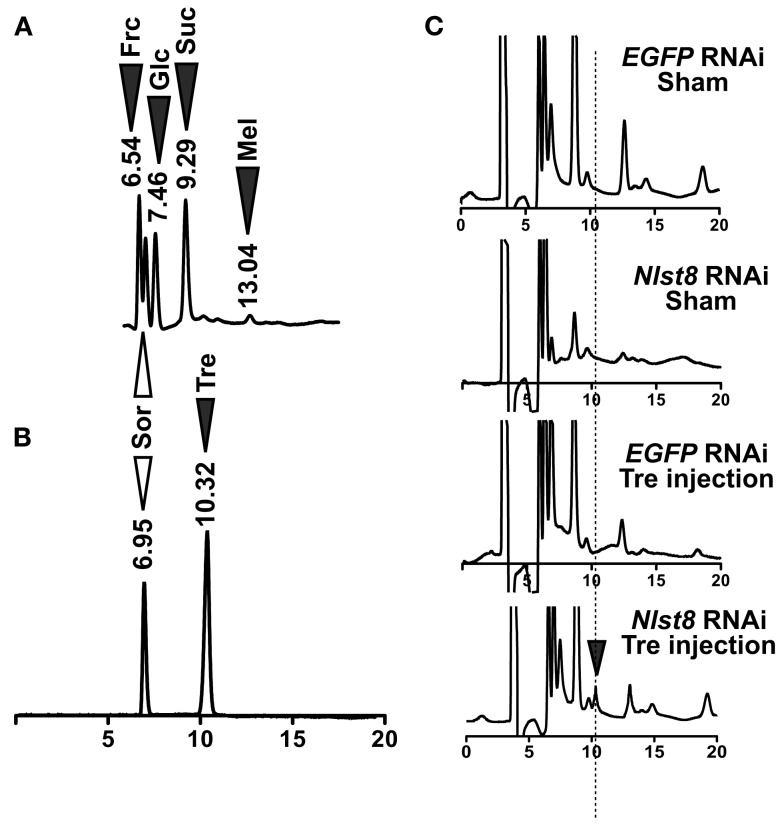
**Detection of sugars in BPH honeydew**. **(A)** HPLC chromatogram representing the sugars present in BPH honeydew. White arrowhead shows sorbitol as an internal standard control; black arrowheads show the sugars detected in honeydew. Frc: fructose, Sor: sorbitol, Glc: glucose, Suc: sucrose, Mel: melezitose. **(B)** Retention time of trehalose and sorbitol. **(C)** Trehalose detection in BPH honeydew. Black arrowhead represents the trehalose (Tre) peak.

Like other phloem-sap-feeding insects, BPH excretes sugar-rich waste as honeydew (Sogawa, 1982). To determine whether honeydew contains trehalose, we analyzed the sugars in BPH honeydew by using HPLC. Trehalose was not detected in the honeydew (Figure A4A in Appendix), indicating that the trehalose in hemolymph is not excreted through the Malpighian tubules to the honeydew. However, trehalose could leak from the hemolymph to the tubule lumen side and be effectively reabsorbed into the Malpighian tubule principal cells. To test this possibility, we investigated whether NlST8 was involved in trehalose reabsorption from the lumen via a proton gradient. Figure 4A illustrates a schema of the experimental design. Injection of *Nlst8* double-strand RNA (dsRNA) into the hemocoel of BPH silenced *Nlst8* gene expression at 48 h after injection (Figure 4B). To create a hyperglycemic state, we injected highly concentrated trehalose, or water as a control, into the hemocoel of BPH pre-treated with either *Nlst8*-dsRNA or *EGFP1*-dsRNA (control). The injection of trehalose boosted the trehalose content in the hemolymph at 30 min after injection, whereas water injection caused no change in the trehalose content. Twenty-four hours after injection of high-concentration trehalose, trehalose content in the hemolymph returned to normal levels owing to the homeostasis (Figure 4C). By using these treated BPHs, we examined whether trehalose was detectable in their honeydew under hyperglycemic conditions. Trehalose was significantly detected in the honeydew of the *Nlst8* RNAi BPH when the high-concentration trehalose was injected (*P* = 0.0023), whereas no trehalose was excreted into the honeydew in *EGFP* RNAi BPH even when the high-concentration trehalose was injected (Figure 4D; Figure A4C in Appendix). Trehalose was also not detectable in the honeydew of the sham controls (Figure 4D; Figure A4C in Appendix). These results show that gene knockdown of *Nlst8* by RNAi disrupts trehalose reabsorption in Malpighian tubules. Taken together, these data suggest that NlST8 genetically regulates trehalose reabsorption in Malpighian tubules.

## Discussion

**Figure A5:**
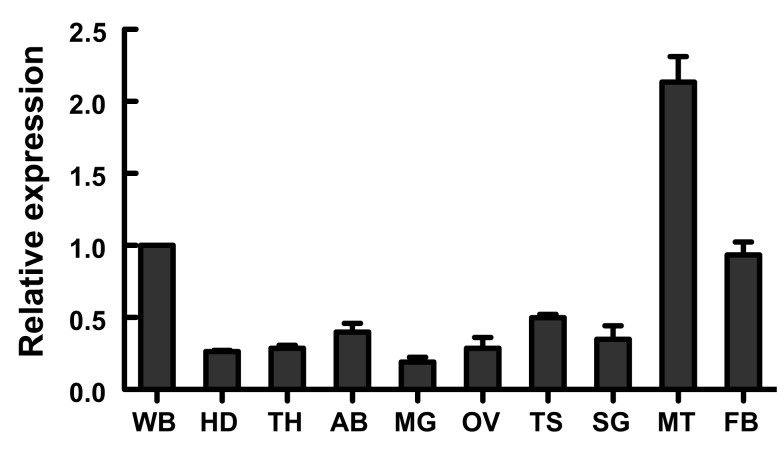
***Nlst8* gene expression by using realtime RT-PCR (modified from Figure 1 in Kikuta et al., 2010)**. Tissues were independently prepared three times. *RP-L4* was used for normalization. Tissue abbreviations: WB, whole-body; HD, head; TH, thorax; AB, abdomen; MG, midgut; OV, ovary; TS, testis; SG, salivary glands; MT, Malpighian tubules, and FB, fat body. Error bars represent the standard deviation.

Previously, we reported the cloning of several sugar transporter genes from the BPH *N. lugens* (Kikuta et al., 2010). Here, we found that one of those genes, *Nlst8*, encodes a membrane protein with trehalose transport activity that is driven by proton (H^+^) electrochemical membrane potentials, indicating that NlST8 is an H^+^-trehalose co-transporter, H-TRET. Spatial expression analysis showed that *Nlst8* is principally expressed in Malpighian tubules (Figure A5 in Appendix), suggesting that NlST8 is involved in trehalose reabsorption in these tubules. The occurrence of sugar reabsorption from the lumen to the Malpighian tubule principal cells in insects has been observed physiologically (Knowles, 1975). However, the molecular context underlying this reabsorption has thus far been obscure. By using an RNAi gene-silencing technique, we demonstrated TRET1/NlST8’s participation in trehalose reabsorption in Malpighian tubules.

Malpighian tubules are excretory tissues in insects and comprise a single layer of squamous epithelial cells adhered intercellularly with septate junctional complexes (O’Donnell, 2008; Beyenbach et al., 2010). Excretion occurs through the tubules by transcellular transport and paracellular transport pathways (Beyenbach et al., 2010). In the former pathway, molecules, absorbed via transporters and/or channels in the basolateral membrane of the principal cells, are actively discharged into the tubule lumen through other transporters or channels situated in the apical membrane. In the latter pathway, molecules are sluggishly excreted along the cleft between the septate junctions. Cations, such as Na^+^ and K^+^, are excreted transcellularly, whereas uncharged small molecules, including polyethylene glycol and sucrose, are discharged paracellularly (Beyenbach and Piermarini, 2011). Trehalose likely seeps passively from the hemolymph into the tubule lumen via the paracellular transport pathway.

In the apical membrane of tubule principal cells, a V-type H^+^ ATPase energizes proton-dependent secondary active transporters by forming an H^+^ gradient (Wieczorek et al., 2009). Cation/*n*H^+^ antiporters, which transport excess Na^+^ and/or K^+^ from the cytosol of the principal cells into the lumen, are representative examples of such transporters. Trehalose uptake by H-TRET1/NlST8 was driven by a proton gradient across the membrane (Figure 2B), suggesting that H-TRET1/NlST8 cooperates with a V-type H^+^ ATPase that probably acts as “the trehalose pump” to promote trehalose reabsorption from the lumen. This idea is supported by the knockdown of *Nlst8*, which led to trehalose excretion into honeydew (Figure 4). Trehalose incorporated into the principal cells must be utilized as an energy source to promote the V-type H^+^ ATPase activity because in insects, the tubules express high levels of soluble trehalase (Derr and Randall, 1966; Dahlman, 1970), which facilitates the degradation of trehalose into glucose.

Recently, another disaccharide transporter, SCRT, was identified from *D. melanogaster*. SCRT appears to be involved in sucrose uptake in the intestinal tract, especially in hindgut (Meyer et al., 2011). Similarly to H-TRET1/NlST8, SCRT exerts H^+^ co-transport activity for disaccharides. The primary structure of SCRT is distinct from that of the TRET1 family, including H-TRET1/NlST8 (Figure A1 in Appendix). Indeed, SCRT closely resembles the mammalian solute carrier family 45 (SLC45; Meyer et al., 2011), whereas the insect TRET1 family belongs to the SLC2 family (Kanamori et al., 2010). For SCRT, trehalose would be a competitive inhibitor for sucrose transport activity, suggesting that trehalose may also be a substrate for SCRT (Meyer et al., 2011). The involvement of SCRT in trehalose reabsorption in the intestinal tract remains obscure.

**Figure 5:**
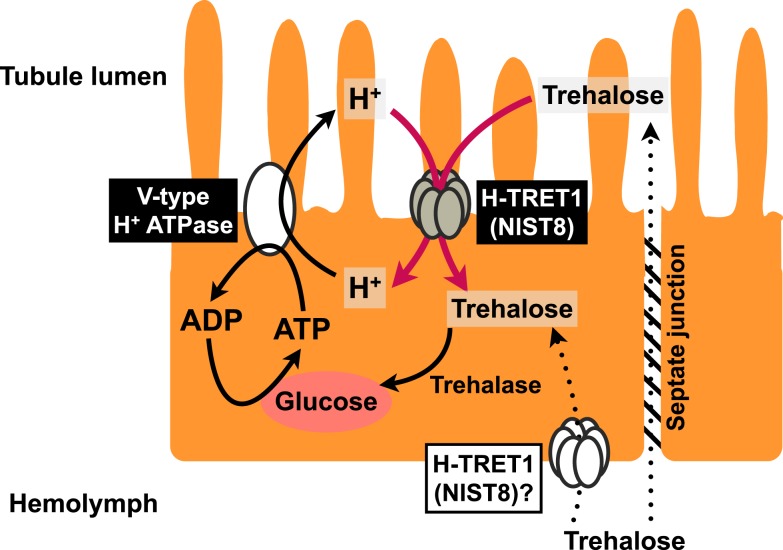
**Schematic model of trehalose reabsorption**. Principal cells mediate proton transfer, driven by a V-type H^+^ ATPase, with H-TRET1 (NlST8) putatively expressed in the apical membrane. Trehalose in the hemolymph that leaks via the paracellular transport pathway through the septate junction would be reabsorbed by H-TRET1 driven by the H^+^ electrochemical membrane potentials from the tubule lumen to the principal cells. This trehalose would then be hydrolyzed into two glucose molecules by trehalase. Glucose would be utilized as an energy source for the proton transfer driven by the V-type H^+^ ATPase.

We conclude that Malpighian tubules use a V-type H^+^ ATPase to power not only the transepithelial secretion of electrolytes but also reabsorption of passively secreted trehalose from the tubule lumen by means of H-TRET1/NlST8 (Figure 5), although the subcellular localization of H-TRET1/NlST8 in the principal cells of the Malpighian tubules has yet to be investigated. Indeed, involvement of tissue other than the tubules in the reabsorption of trehalose remains controversial. Further histochemical, cytochemical, and genetic analyses using transgenic and knockout insects will uncover the fine details of the molecular basis of trehalose reabsorption via H-TRET1/NlST8.

## Materials and Methods

### Insects and plants

BPH (strain: Izumo) were reared and maintained on rice seedlings at 26°C with 16 h light: 8 h dark periods. The rice plants for honeydew collection were cultivated at 25°C.

### RNA isolation and cDNA cloning

Total RNA was isolated from young female adults by using the RNeasy Mini kit (Qiagen, Hilden, Germany). *Nlst8* cDNA sequence analysis was performed with an ABI prism 3730 and a BigDye Terminator v3.1 cycle sequecing kit (Life Technologies, Carlsbad, CA, USA). Sequence data were analyzed with GENETYX-MAC ver. 16 software (GENETYX, Tokyo, Japan).

### Synthesis of capped RNA

An expression vector for the BPH sugar transporter gene *Nlst8* was constructed from PCR products amplified with specific primers containing restriction sites. The PCR products were cloned into the pT7XbG2 (DDBJ accession number, AB255037) vector digested with the restriction enzymes *Bgl*II and *Eco*RV. To determine NlST8 localization in *Xenopus* oocytes, *Nlst8* was cloned into the pT7XbG2-AcGFP1 (DDBJ accession number, AB255038) vector digested with the restriction enzymes *Bgl*II and *Eco*RV. *Nlst8* PCR products were amplified with specific primers containing the appropriate restriction sites. The primers used are listed in Table A1 in Appendix. The template DNA for cRNA synthesis was amplified with high-fidelity DNA polymerase (KOD plus, TOYOBO, Osaka, Japan) from the expression vectors by using primers containing the T7 and T3 promoters. The *Nlst8* cRNAs were obtained by using the mMESSAGE mMACHINE T7 kit (Life Technologies) according to the manufacturer’s standard protocols.

### Expression of the NLST8 transporter in *Xenopus* oocyte membranes and quantification of sugar transport activity by using HPLC

**Figure A6:**
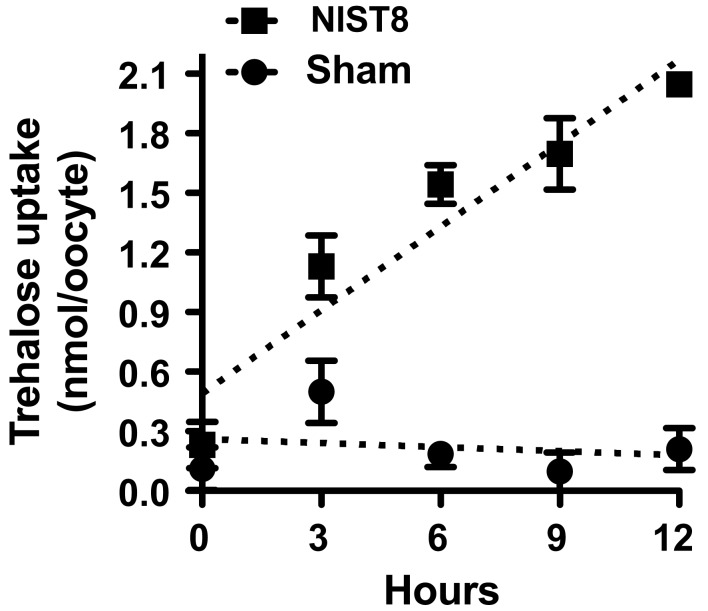
**Time course of trehalose uptake in NlST8-expressing *Xenopus* oocytes**. Ten oocytes were analyzed in each assay; error bars represent the standard error (*n* = 3). Sham-treated oocytes served as a control. The trehalose concentration in the buffer was 105 mM.

The methods were based on those used in our previous report (Kikawada et al., 2007). Briefly, the cRNA was injected into *Xenopus* oocytes, and the oocytes were incubated in modified Barth’s saline (MBS) buffer, pH 7.8, for 3 days at 20°C. The oocytes were incubated with various sugars at a concentration of 105 mM in MBS buffer at 20°C for 6 h, and then washed three times with sterilized MBS. Actually, trehalose uptake by NlST8-expressing oocytes was markedly increased after 12 h of incubation compared with the control (Figure A6 in Appendix). Sugar incorporated into the oocytes was measured by means of HPLC with an HPX-87C column (Bio-Rad, Hercules, CA, USA). Oocytes injected with water served as a negative control to evaluate endogenous sugar uptake by the oocytes. To examine the effect of Na^+^ on NlST8 sugar uptake, the injected oocytes were incubated in Na^+^-free buffer. To prepare the Na^+^-free buffer, 90.4 mEq/L of Na^+^ in MBS buffer was replaced with equivalent concentration of choline chloride. The pH dependency of the sugar transport activity of NIST8 was investigated by using MBS buffer containing MES or Tris at pH 5.0–6.0 or 7.8–9.0, respectively. For the kinetic analysis, trehalose uptake into oocytes was observed over a range of concentrations from 0 to 200 mM at 20°C. The *K*_m_ and *V*_max_ values were calculated by using Prism 5 (GraphPad Software, La Jolla, CA, USA). For statistical analyses, Student’s *t*-test or one-way ANOVA before Tukey’s multiple comparison tests were also performed by using Prism 5 (GraphPad).

### AcGFP1 localization in the *Xenopus* oocyte cellular membrane

After cRNA or water injections, *Xenopus* oocytes were incubated for 3 days at 20°C. They were subsequently embedded in Tissue-Tek^®^ OCT™ compound (Sakura Finetek Japan, Tokyo, Japan), and stored at −80°C until use. Frozen sections with a cryostat (CM 3050S, Leica, Bensheim, Germany) were observed by means of fluorescent microscopy (Leica DMR) under UV light.

### Hyperglycemic insect

To create a hyperglycemic state, ca. 50 nl of either 1 M trehalose or distilled water as a negative control was injected into the segment between the thorax and the abdomen of BPH female by using a capillary injector (Eppendorf AG, Hamburg, Germany).

### Measurement of BPH hemolymph sugar

BPH female adults, collected within 24 h of emergence, were fed on rice plant seedlings for 24 h. At least 10 BPH were used to collect the hemolymph for each assay and were preserved on ice until use. Biological samples were prepared three times independently. The method used to collect the BPH hemolymph was modified from that previously published (Moriwaki et al., 2003). The foreleg of the BPH was torn off with forceps at 20°C and the hemolymph was poured into a capillary (World Precision Instruments, Inc., Sarasota, FL, USA). Sugar content was determined by using HPLC with an HPX-87C column (Bio-Rad; Watanabe et al., 2002).

### BPH honeydew collection and quantification

BPH female adults, collected within 24 h of emergence, were fed on rice plants at the 3–5-leaves stage for 24 h covered with a Parafilm sachet (Pathak et al., 1982). Honeydew excreted into the sachet was collected to evaluate its sugar content by using HPLC with an NH_2_ column (CAPCELL PAK NH_2_, 250 mm × 4.6 mm ID, SHISEIDO, Tokyo, Japan). Elution was performed with H_2_O:CH_3_CN (25:75 v/v) at a flow rate of 0.8 ml/min at 35°C.

### RNAi

Partial sequences of genes for *Nlst8* (508 bp) and *EGFP* (420 bp) were amplified with each specific primer described in Table A2 in Appendix, and then cloned into the pGEM-T vector. To produce double-strand RNA (dsRNA) for the corresponding genes, we used the T7 Ribomax Express RNAi System (Promega, Madison, WI, USA) according to the manufacture’s instruction manual. In particular, PCR products amplified by either of the specific primers (Table A2 in Appendix) fused with the T7 promoter sequence (5′-TAATACGACTCACTATAGG-3′) at the 5′-end were used as templates for dsRNA synthesis by *in vitro* transcription with T7 RNA polymerase. BPH female adults, collected within 24 h of emergence, were injected in the segment between the thorax and the abdomen with approximately 50 nl of dsRNA solution (1 μg/μl) in RNase-free water, by using a capillary injector (Eppendorf AG). To examine *Nlst8* gene expression, quantitative RT-PCR was performed by using the SYBR Green I Master Mix (Roche Diagnostics, Basel, Switzerland) with a LightCycler 480 (Roche Diagnostics) after total RNA isolation and first-strand cDNA synthesis (TaKaRa, Shiga, Japan). The ribosomal protein L4 gene (*RP-L4*) was used for normalization. Primers for *Nlst8* and *RP-L4* are described in Table A3 in Appendix.

## Conflict of Interest Statement

The authors declare that the research was conducted in the absence of any commercial or financial relationships that could be construed as a potential conflict of interest.

## Appendix

**Table A1:** **Primers for cRNA construction**.

Gene	Forward primer	Reverse primer
*Nlst8*::*AcGFP1*	5′-GTCG**AGATCT**CCACCATGAAGCGATTGCTGCGTGC-3′	5′-AGCTC**GATATC**AATGGAGCTTGGAAGCGGTTTG-3′
*Nlst8*	5′-GTCG**AGATCT**CCACCATGAAGCGATTGCTGCGTGC-3′	5′-AGCTC**GATATC**CTAAATGGAGCTTGGAAGCGGTTTG-3′

*Boldface denotes the *Bgl*II (forward) and *Eco*RV sites (reverse); underline shows the Kozak sequence*.

*Primers for the *AcGFP1* and *PvTret1* cRNA constructions were previously described (Kikawada et al., 2007; Kikuta et al. 2010)*.

**Table A2:** **Primers for dsRNA construction**.

Gene	Forward primer	Reverse primer
*Nlst8*	5′-TGGTCACAGGTTTTGGCGGCT-3′	5′-GAACTGGTATAGCTGCTCCTAGC-3′
*EGFP*	5′-AAGTTCAGCGTGTCCGGCGA-3′	5′-GAAGTTCACCTTGATGCCGTT-3′

**Table A3:** **Primers for quantitative RT-PCR analysis**.

Gene	Forward primer	Reverse primer
*Nlst8*	5′-TTATTGGAGAGATTAAGTTAC-3′	5′-TTTGCGAATAAAATAATTAAACTAC-3′
*Rp-L4*	5′-TTCGTCATCTGGACCCAATCGG-3′	5′-AACTCCCTTCTGTGGCGGTTTGA-3′

## Abbreviations

2-DOG, 2-deoxy-d-glucose; BPH, brown planthopper; CCCP, carbonyl cyanide *m*-chlorophenyl hydrazone; cRNA, capped RNA; EST, expressed sequence tag; GLUT, glucose transporter family; MBS, modified Barth’s saline; MES, 2-(*N*-morpholino)ethanesulfonic acid; MFS, major facilitator superfamily; SLC2, solute carrier family 2; TRET1, trehalose transporter 1
